# Abatement of radioiodine in aqueous reprocessing off-gas

**DOI:** 10.3389/fchem.2022.1078668

**Published:** 2023-01-11

**Authors:** Allison T. Greaney, Randy O. Ngelale, Stephanie H. Bruffey, Leigh R. Martin

**Affiliations:** ^1^ Oak Ridge National Laboratory, Oak Ridge, TN, United States; ^2^ Ultra Safe Nuclear Corporation, Oak Ridge, TN, United States

**Keywords:** aqueous reprocessing, radioiodine, silver mordenite, off-gas, organic iodide

## Abstract

The reprocessing used nuclear fuel (UNF) releases volatile fission and activation products, including ^129^I, into the off-gas of a processing plant. Mitigation of the release of vapor phase radionuclides is necessary for meeting regulatory requirements in the United States and other countries. In an aqueous reprocessing plant, volatile radioiodine could be present in several forms, depending on the chemistry of the process used. Inorganic iodine will be the predominate species in any shearing or voloxidation pretreatment off-gas and dissolver off-gas (DOG). Organic iodides such as CH_3_I, C_4_H_9_I, and C_12_H_25_I have been proposed to be generated during solvent extraction; thus, these species must be captured from the vessel off-gas (VOG). The abatement of inorganic and organic iodide species to meet United States regulatory requirements has been demonstrated in laboratory experiments using Ag-based solid sorbents. The data presented in this paper includes the effect of gas composition (e.g., the presence of water vapor and NO_*x*_), iodine speciation (I_2_, CH_3_I, C_4_H_9_I, C_12_H_25_I), and sorbent bed parameters (e.g., temperature, sorbent age) on complete iodine capture on Ag-mordenite in an aqueous reprocessing plant.

## 1 Introduction

### 1.1 EPA regulatory limits on radioiodine release

The abatement of radioiodine from a nuclear fuel reprocessing facility in the United States would be governed by two regulatory bodies: the United States Environmental Protection Agency (EPA) and the United States Nuclear Regulatory Commission (NRC, 2012). Of these two regulatory bodies, EPA regulations impose stricter limits on the amount of iodine potentially released from any proposed facility. Under 40 CFR Part 190 (EPA-1, 1977), *Environmental Radiation Protection Standards for Nuclear Power Operations*, the total iodine release to the environment from the entire fuel cycle must be no greater than 0.005 Ci/GWy of ^129^I of electrical energy generated *via* the fuel cycle. A framework for meeting the 0.005 Ci/GWy for ^129^I, stipulated in 40 CFR 190 that also accounts for variations in source terms as a function of fuel burnup, was described in Jubin et al. (2012).

### 1.2 Iodine partitioning in an aqueous reprocessing facility

**FIGURE 1:**
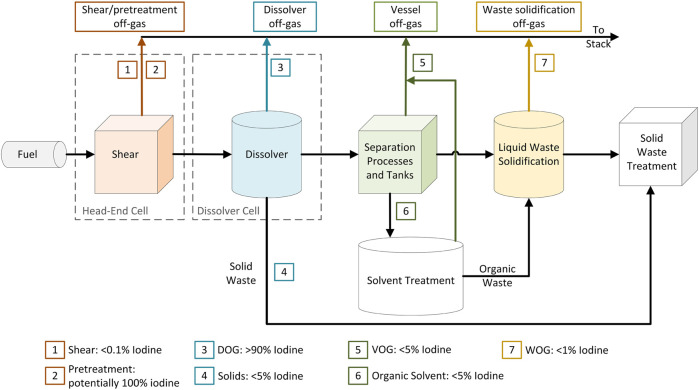
Diagram of an aqueous reprocessing facility with estimated iodine partitioning through the plant expressed as a percentage of the total initial used nuclear fuel (UNF) iodine inventory.

The general scheme of aqueous reprocessing plants follows a sequence of steps involving the shearing of fuel pins that have been stored between 3 and 5 years, the dissolution of the fuel in acidic media, separation of constituents using select partially immiscible organic extractants, such as tributyl phosphate (TBP) in an organic diluent such as kerosene, followed by product and waste stream treatment (Figure 1). It is estimated that >0.1% of radioiodine is released in the shearing step with a further >90% being released during the dissolution of the fuel. The remaining 5%–10% is carried into the solvent extraction steps.

For used fuel with a burn up between 30 and 60 GW days per metric ton in heavy metal (GWd/tIHM), Jubin et al. estimate ^129^I activities of 0.92 and 0.90 Ci/GWy. This necessitates decontamination factors of 184 and 179 respectively. To meet these decontamination factors, a more thorough investigation into the specifics of ^129^I partitioning in a fuel reprocessing plant is required.

Jubin and Strachan (2015) estimated an initial iodine inventory of 368 g/tIHM based on simulations for fuel burnup of 60 GWd/tIHM after 5 years of cooling. Of this quantity, the bulk is expected to be observed in the shearing and dissolution process off-gas stream (i.e., DOG) (∼347g/tIHM) with the remainder observed in the solvent extraction step (i.e., vessel off-gas [VOG]) (∼10.7 g/tIHM) (Hebel and Cottone, 1982).

The forms and species of iodine -bearing compound relevant to aqueous reprocessing systems were investigated in Bruffey et al. (2015). By using enthalpy, entropy and heat capacity modeling, and free energy minimization, several thermodynamically favorable reaction pathways were identified based on organic and inorganic species found in reprocessing systems.

### 1.3 Organic speciation of iodine

Organic iodides likely form during solvent extraction, which may partition into the VOG. Degradation of tributyl phosphate and the organic diluent due to acid hydrolysis reactions and radiolysis yields shorter chain organic and radical species. Subsequent attacks by these radical species producing further short chain organics has been identified by Mincher et al. (2009), these species may react with iodide in the system to form organic iodides. Iodides of straight chain alkanes ranging from methyl to dodecyl were found during tests at the Wiederaufarbeitungsanlage Karlsruhe (WAK) (Herrmann et al., 1988). Dodecyl iodide has been reported as the most prevalent organic iodide species under process conditions in the VOG (Heinrich et al., 1981; Herrmann et al., 1988). The recycling of nitric acid streams results in the introduction of organic impurities from solvent extraction stages, into the dissolution stage promoting the formation of short chain organic iodides that end up in the DOG. Further, organic impurities have been shown to be present in commercially acquired nitric acids that are likely to be used in commercial scale activities. Nakamura et al. (1973).

### 1.4 Industrial methods of iodine capture

Several different methods have been proposed and implemented to varying degrees industrially to facilitate iodine capture from the off-gas. Among them, two major categories (solid adsorbent methods and wet scrubbing methods) exist. Wet scrubbing is done either as a caustic solution of 1–2 M NaOH (McKay, 1982) (Table 1). In cases of significant CO_2_ presence, KOH may be used to prevent sodium carbonate precipitation (Dean, 1999). Caustic scrubbing yields high efficiency for elemental iodine but poor efficiency for organic forms (Trevorrow et al., 1983). Alternatively, the Mercurex process, consisting of a scrub solution of 0.4 M Hg(NO_3_)_2_ and up to 14 M HNO_3_, yields higher removal efficiency for organic forms (Jubin, 1988). A number of silver based solid adsorbents have been found favorable for industrial use either alone or in conjunction with wet scrubbing techniques (McKay, 1982). For a complete review of iodine sorbents used historically, see Riley et al. (2016).

**TABLE 1:** Iodine capture methods at various plants.

Plant	Location	Proposed method	References
Hanford WTP	Washington (United States)	Caustic scrub, Silver-loaded zeolite	Hebel and Cottone (1982)
AGNS	Barnwell, SC (United States)	Mercurex, Silver-loaded faujasite	Jubin (1988)
TBP	Windscale (United Kingdom)	Caustic scrub	Jubin (1988)
WAK	Karlsruhe (Germany)	AC 6120 (silver based adsorbent)	Wilhelm and Furrer (1977)
UP_2_	La Hague (France)	Caustic scrub	Jubin (1988)

Reduced silver mordenite, the sorbent used in this study, has been researched as an iodine sorbent since the 1970s and has been demonstrated to effectively capture organic iodide (as CH_3_I) in addition to inorganic I_2_ (e.g., Thomas et al., 1978; Scheele et al., 1983; Nenoff et al., 2014).

### 1.5 Study objective

This paper presents a comprehensive overview of proposed methodologies for iodine capture from aqueous reprocessing off-gas using data collected through several sets of experiments that explored the generation and capture of organic iodides on solid sorbents. In order to meet EPA and NRC regulations, iodine abatement tests were designed to explore the effects of gas stream chemistry (i.e., varying [NO], [NO_2_], and [H_2_O]), iodine speciation (i.e., iodoalkanes vs inorganic iodine), and sorbent bed engineering design (i.e., flow rate, sorbent age, and sorbent bed temperature) on iodine capture by Ag mordenite. The results are presented by off-gas stream with a particular focus on the DOG, which will likely contain >90% of the iodine inventory, and the VOG, which could contain ∼5% of the iodine inventory.

## 2 Iodine speciation experiments

To confirm the potential speciation of organic iodides expected in DOG and VOG streams, simple bench-top experiments simulating dissolver and solvent extraction conditions have been conducted. Processes were simulated in a 500 ml round-bottom flask held in a heating mantle. Air flowed into the flask at ∼0.1 LPM and bubbled through the process liquid. Then, the off-gas generated in the headspace was sampled with a gas-tight syringe. Off-gas samples were analyzed in 200 ml volumes with an Agilent 8890 Ga Chromatograph (GC) coupled to a 5977B mass spectrometer. A 30 m, 0.25 mm inner diameter, DB-UI GC column was used with a temperature gradient of 30°C–200°C and a ramp time of 10°C/min. This method captured organic iodide standards ranging from CH_3_I to C_12_H_25_I. Peaks in the chromatogram were positively identified using their mass spectrum coupled to a NIST database in the MassHunter Software. These experiments provide the relative abundances of organic iodides in the headspace samples. The measurements are by gas phase standards, however these data cannot be used to calculate an extract concentration because of peak interferences from other volatile organics present in the headspace.

In the DOG simulations, the process liquid comprised 100 ml of 3M HNO_3_ that was previously contacted and separated from 100 ml of 30 vol% TBP/70 vol% n-dodecane mixture to represent recycled HNO₃ in a dissolver. The acid was heated to 100°C, and 1 ml of 10% KI solution was added to the flask (∼1,000 ppm I^−^). This iodine concentration is elevated above expected dissolver conditions but was chosen such that iodine speciation in the off-gas would be easily detectable. In VOG simulations, the process liquid comprised 50 ml of 30 vol% TBP/70 vol% n-dodecane mixture and 50 ml of the residual HNO₃ solution left over from the dissolver experiment. This aqueous-organic mixture was maintained at 40°C and mixed vigorously throughout the experiment. Off-gas samples were collected over 6 h during both experiments.

The DOG benchtop experiments primarily detected I_2_, followed by roughly equivalent concentrations of CH_3_I and C_4_H_9_I (∼1 ppm each). The presence of minor C_4_H_9_I was unexpected given prior focus on I_2_ and CH_3_I in the DOG literature. This alkyl iodide likely formed only in the experimental DOG because recycled acid containing TBP was used instead of fresh acid. Butyl iodide is not expected to form in conditions where fresh acid is used during dissolution, but CH_3_I has been proposed to form in fresh-acid experiments because of impurities in the acid (Nakamura et al., 1973). The VOG experiments resulted only in the detection of CH_3_I and C_4_H_9_I in the off-gas. No I_2_ or C_12_H_25_I was detected in the VOG. Although C_12_H_25_I may have formed in solution, its low vapor pressure likely precluded it from forming in any considerable quantity within the headspace.

## 3 Iodine abatement experiments

### 3.1 Sorbent selection

Silver-functionalized sorbents are considered the industry standard given the thermodynamically favorable reaction of Ag and iodine to form AgI or AgIO_3_ under most modeled off-gas capture conditions. These sorbents include Ag-mordenite (AgZ), Ag-faujasite (AgX), Ag-alumina (AgA), and Ag-aerogel (Routamo, 1996). Of these sorbents, AgZ is physically and chemically robust to NOx gasses in the DOG, given its high Si to Al ratio, while still maintaining a relatively high capacity for iodine. Thus, all experiments presented here reflect iodine adsorption onto AgZ.

Silver mordenite is procured from IONEX and contains 9.5–11.9 wt% Ag. The AgZ is reduced to Ag^0^Z in-house under a 4% H_2_/Ar gas stream, which significantly improves its iodine loading potential. The sorbent pellets are approximately 1.6 x 3 mm, with a bulk density of 1.87 g/cm^3^, surface area of 179 m^2^/g, and chemical formula of Ag_4.09_H_4.12_(AlO_2_)_8.21_(SiO_2_)_43.26_ ·*x*H_2_O (Nan, 2017). The chemical and physical effects of sorbent aging in off-gas streams have been well characterized (Wren et al., 1986).

The silver in the mordenite chemically reacts with iodine to form AgI. This compound is stable under most industrial conditions, therefore AgZ likely not suitable for regeneration. Jubin et al. (2019) attempted regeneration of iodine-loaded AgZ and AgNO_3_-impregnated alumina at 200°C and found that less than 1% of the adsorbed iodine was released over 3 h.

### 3.2 Experimental methods

**FIGURE 2:**
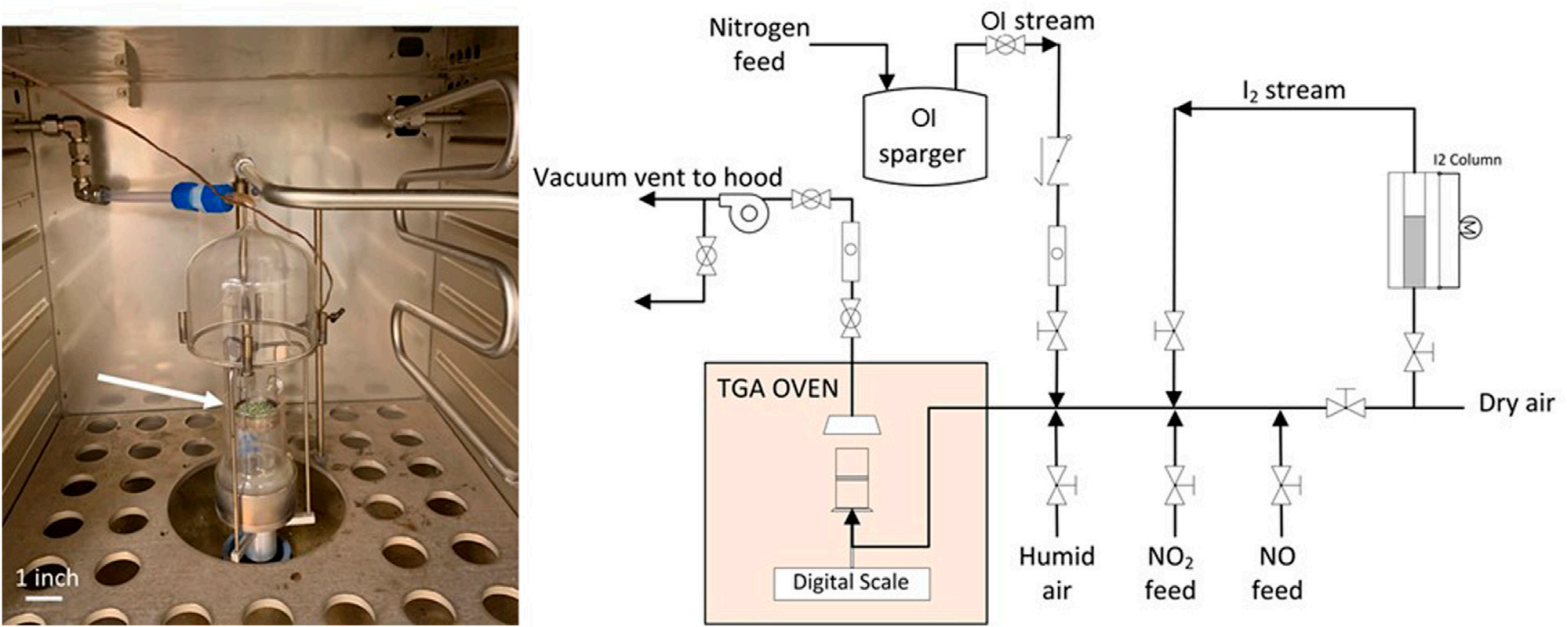
Photograph of the TGA thin bed that contains AgZ pellets that reacted with iodine to form AgI or AgIO_3_ (green pellets at end of arrow) (left); schematic of the manifold used for TGA testing at ORNL (right). OI (right figure) represents organic iodide spargers.

A thermogravimetric analyzer has been used to determine iodine mass gain on AgZ. A custom-manifold upstream of the TGA allows various components to be valved-in to the simulated off-gas stream (Figure 2). This includes NO_*x*_ gasses (NO + NO_2_), humid air, and iodine species (i.e., I_2_, CH_3_I, C_4_H_9_I, or C_12_H_25_I). All flow rates are regulated with Sierra Mass Flow controllers that display the active flow rate for monitoring. Nominal tests run at a total superficial velocity of 10 m/min at 150°C. Apparent mass gain measured with the TGA is confirmed with neutron activation analysis (NAA) at the High Flux Isotope Reactor at Oak Ridge National Laboratory. Detailed methods can be found in other works (Greaney et al., 2020; Greaney et al., 2021).

Gaseous iodine is generated by flowing a known flow rate of air through a packed bed of crystalline I_2_ held at 18°C, then diluting the I_2_-saturated gas stream into a larger gas flow to a set concentration. Vapor phase organic iodides are generated with the same concept but with liquid CH_3_I, C_4_H_9_I, and C_12_H_25_I bubblers held constant at ‒30°C, 20°C, and 75°C, respectively. Nitric oxide is generated with a compressed cylinder, and NO_2_ is generated by heating a cylinder of liquid N_2_O_4_ to generate NO_2_ at a set flow rate. Water vapor is added to the gas streams with a water bubbler.

## 4 DOG iodine capture

### 4.1 DOG experimental design

In a nominal aqueous reprocessing flow sheet, the DOG is expected to contain >90% of the iodine present in the used nuclear fuel (Sakurai et al., 1989). This iodine is predominately in inorganic forms, such as I_2_ (Sakurai et al., 1989; Sakurai et al., 1997), but minor CH_3_I and C_4_H_9_I may form, as shown thermodynamically (Bruffey et al., 2015) and experimentally in this study. The DOG contains other chemical components that could affect iodine sorption onto Ag-based sorbents, mainly water and NO_*x*_ gasses. The DOG is a humid gas stream with a dew point potentially >20°C, depending on the configuration of condensers upstream of the iodine sorbent bed. Additionally, heating HNO₃ will form NO_2_ and NO gas, and concentrations will likely range between 0.5% and 1.5% total NO_*x*_ (Birdwell 1990).

To test the effects of water and NO_*x*_ on iodine sorption onto AgZ, a test matrix of eight experiments was designed using a fractional factorial analysis scheme for both I_2_ and CH_3_I following the study performed in Jubin (1981). Four variables were explored: sorbent bed temperature of 135°C or 165°C, dew point of ‒70°C or 0°C, NO_2_ concentration of 0% or 1%, and NO concentration of 0% or 1%. These temperatures were chosen to bracket the optimized operating temperature of 150°C to determine if slightly adjusting the temperature could increase iodine sorption. The water concentrations were chosen to compare an extremely dry system to the humid stream expected in the DOG. The NO and NO_2_ gas concentrations were selected from the experimental results of Birdwell (1990) that show the NO + NO_2_ concentration in the off-gas typically fluctuate between 0% and 1.5%, after the gas is scrubbed with two condensers.

**FIGURE 3:**
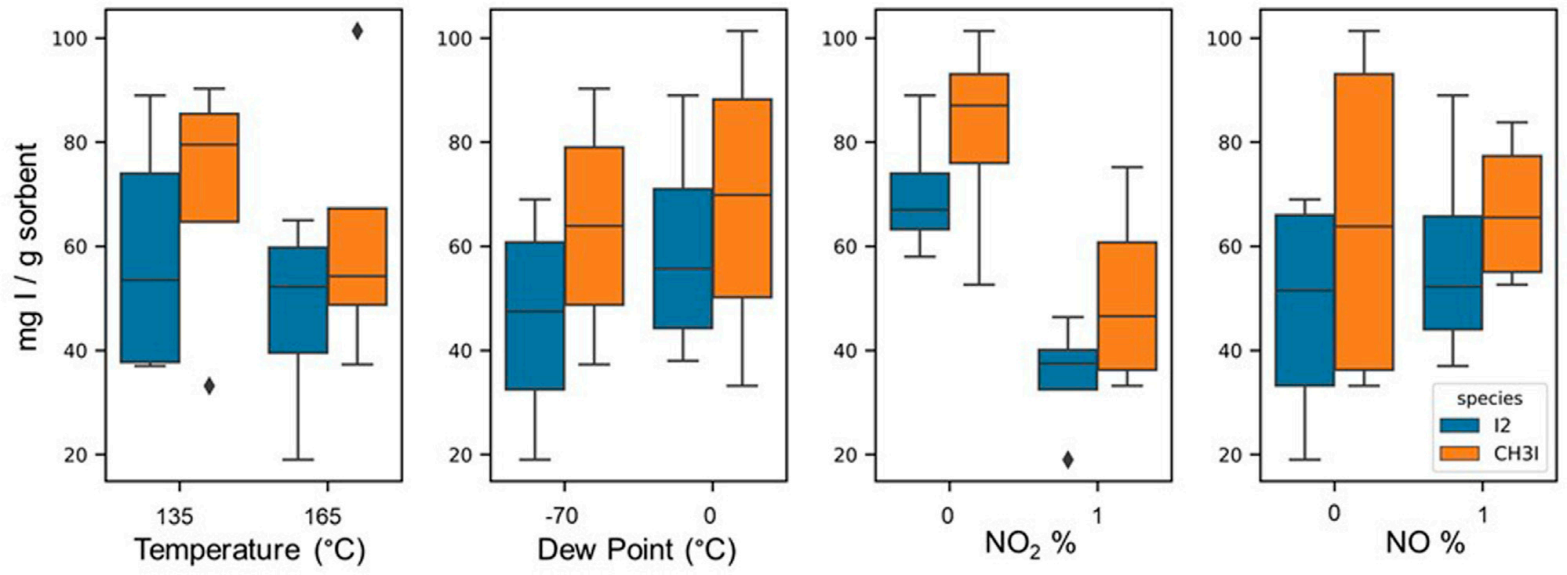
Effects of each temperature, dew point, NO_2_ vol%, and NO vol% on I_2_ and CH_3_I sorption (mg I/g sorbent) on AgZ. Each box and whisker plot represents iodine loading results of four tests. Iodine data are in blue (left-hand boxes), and CH_3_I data are in orange (right-hand boxes).

Vapor phase I_2_ (25 ppm-mol) was flowed over a thin bed of AgZ. Mass gain was measured in real time using a TGA, and iodine loading was confirmed with NAA. Tests were run until loading was complete (i.e., when the TGA loading curve did not show any mass gain for at least 24 h). This typically occurred in 1–2 weeks. The results of these tests are presented as box and whisker plots in Figure 3 in which iodine loading is measured in milligrams of iodide per Gram of sorbent (*y*-axis), and the two set points for each variable are plotted on the *x*-axis. Nominal iodine loading on AgZ (9.5–11.9 wt% Ag) under dry conditions results in ∼100 mg I per g of sorbent (stylized as mg I/g sorbent). Replicate tests suggest that errors on iodine loading experiments vary within 10 mg I/g sorbent.

### 4.2 DOG results

These results indicate that the presence of NO and the dew point of the gas stream do not affect iodine sorption on AgZ in a statistically significant way over ∼2 weeks test durations. Increasing the temperature of the sorbent bed from 135°C to 165°C has a slightly negative effect on both I_2_ and CH_3_I sorption, dropping average loadings to ∼55 mg I/g sorbent. Thus, sorbent beds are recommended to be maintained below 165°C. Nitrogen dioxide has the most detrimental effect, decreasing sorbent capacity by up to 62% with typical loadings between 30 and 50 mg I/g of sorbent. This is likely due to aging effects on the sorbent as the NO_2_ oxidizes the Ag^0^ to Ag^1+^, forming Ag_2_O and decreasing the overall reactive potential for AgI or AgIO_3_ to form. When an equivalent volume percent of NO is added to the system in addition to NO_2_, iodine sorption increased relative to tests with solely NO_2_. This implies that adding NO decreases the effect of sorbent oxidation by NO_2_. Thus, the ratio of NO to NO_2_ in the DOG should be monitored to assess sorbent efficiency potential.

The detrimental effect of NO_2_ on AgZ capacity must be considered when designing flow sheets for sorbent beds in an aqueous reprocessing facility. Although AgZ has a maximum sorbent efficiency of ∼100 mg I/g sorbent, the effect of NO_*x*_ aging will likely lower actual sorbent efficiency to 40–50 mg I/g sorbent. Nevertheless, deep-bed tests completed at Idaho National Laboratory in the presence of NO_2_ show that AgZ sorbent beds can maintain decontamination factors of >10,000 for I_2_ and >1,000 for CH_3_I, even with sorbent capacities decreased to ∼40 mg I/g sorbent (Bruffey et al., 2019). The tests conducted in this paper and at INL suggest that DOG iodine sorbent systems should be conservatively modeled to accommodate 40–50 mg I/g sorbent to meet required decontamination factors.

## 5 VOG iodine capture

### 5.1 VOG experimental design

In a nominal aqueous reprocessing flow sheet, the VOG is expected to contain an estimated 5% of the total iodine inventory. Residual iodine in the dissolver can remain in solution or as AgI or PdI colloids that precipitate out (Figure 1). Any iodine that remains in the aqueous phase will be transferred to the separation stage where it will either partition into the organic phase or be volatilized into the VOG. Here, organic iodides such as CH_3_I, C_4_H_9_I, and C_12_H_25_I have been suggested to form in the off-gas in parts per billion quantities (Jubin et al., 2013; Bruffey et al., 2015). Although the VOG simulation experiments in this study did not detect C_12_H_25_I in the off-gas, it was still included in the AgZ sorption experiments. If present in the VOG, then all these iodine species must be abated to meet United States regulatory standards.

A series of experiments were completed to test the effects of varying organic iodide speciation and concentration in the off-gas, superficial velocity of the off-gas, and effects of aging on AgZ sorbent capacity. For these experiments, ∼2 g of reduced AgZ were loaded into the thin-bed TGA and exposed to gas streams containing 5, 10, or 50 ppm of CH_3_I, C_4_H_9_I, and C_12_H_25_I until the sorbent reached saturation. The time to saturation and total saturation capacity were recorded. Because the VOG stream has a lower number of iodine moles passing through the sorbent beds, beds may stay online in a facility longer than DOG beds. Thus, these experiments were repeated with AgZ that had been aged for 9 months in a humid gas stream to determine how long-term sorbent aging may affect sorbent capacity.

### 5.2 VOG results

**FIGURE 4:**
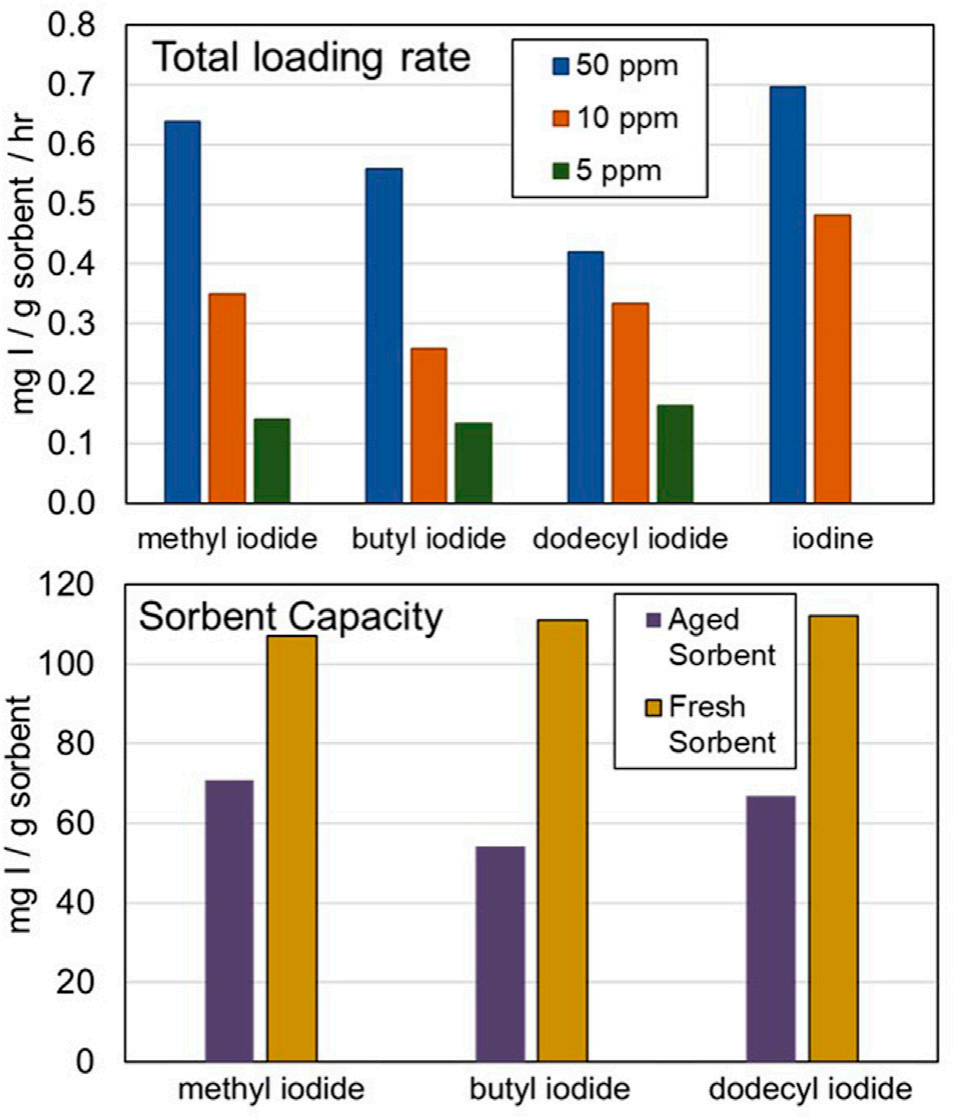
Summary of organic iodide experimental results. (Top) Effects of organic iodide chain length and concentration on total loading rate. (Bottom) Effects of sorbent aging on total sorbent capacity for organic iodide adsorption.

These experiments found that the sorption rate of organic iodides by AgZ depends on the hydrocarbon chain length and the concentration in the off-gas. At 50 ppm in the off-gas, the sorption rate on to fresh AgZ is 8% slower for CH3I, 20% slower for C4H9I, and 40% slower for C12H25I relative to I_2_, which averages 0.70 mg I per g sorbent per hour (Figure 4). The slower loading rate of the longer-chain organics implies that they may be more penetrative into the sorbent bed than I_2_. When 9-month aged sorbent was tested *in lieu* of “fresh” sorbent, sorption rate on to AgZ decreased by 40%–60% for the organic iodides in concentrated (50 ppm) gas streams. At more realistic VOG concentrations (5 ppm), the sorption rate on to fresh AgZ of the three organic iodides is nearly identical: 0.14 mg I/g sorbent/hour ( ± 0.03 mg I/g sorbent/hour). When a 50% reduction in sorption rate due to aging is factored in, this iodine sorption rate may be nearer to 0.07 mg I/g sorbent/hour.

Although the sorption rate varies as a function of hydrocarbon chain length, the saturation concentration does not. The capacity of fresh AgZ ranges from 106 to 112 mg I/g sorbent for the three organic iodides and I. Aging AgZ in a humid air stream for 9 months drops the overall sorbent capacity by ∼35% for CH3I, ∼50% for C4H9I, and ∼40% for C12H25I for an overall iodine capacity of 35–70 mg I/g sorbent. These data mirror the results of the DOG testing, which shows that sorbent that is more quickly aged in a highly oxidizing environment has an iodine capacity between 40 and 50 mg I/g sorbent. Because the VOG stream does not contain the strong oxidant NO_2_, the sorbent likely ages slower relative to DOG sorbent. The reduction in sorbent capacity from ∼100 mg I/g sorbent to ∼50 mg I/g sorbent may occur over longer timescales in the VOG than the day-long timescales observed in the DOG testing. However, these bench-scale tests did not include additional chemical components (e.g., volatilized organics) that could be present in the VOG that could reduce sorbent capacity for iodine due to physiosorption. The effect of these components should be further investigated.

Throughout the long-chain organic iodide testing, C_12_H_25_I was difficult to maintain in the gas phase in the simulated VOG. The vapor pressure of C_12_H_25_I is exceedingly low: 0.7 mm Hg compared with 314 mm Hg for C_4_H_9_I at 100°C (Li and Rossini 1961). Thus, if any point of the VOG is not heat-traced or insulated, there is a high likelihood that C_12_H_25_I will condense out of the gas phase. Herrmann et al. (1988) previously suggested that C_12_H_25_I was the predominant phase in the VOG; however, this phase was measured in a cold trap and not directly in the gas phase. The experiments presented here suggest that C_4_H_9_I and CH_3_I are more prominent in the VOG.

## 6 Conclusion

Multiple experiments were conducted to comprehensively assess the abatement of radioiodine across the DOG and VOG in an aqueous reprocessing facility. These experiments assessed the effects of gaseous components (e.g., NO_*x*_ and humidity in the DOG) and iodine speciation on abatement behavior. The DOG will primarily contain I_2_ and minor CH_3_I and C_4_H_9_I. Although AgZ may have a full capacity of ∼100 mg I/g sorbent, the effect on the sorbent exposed to the chemistry of the DOG and longevity of the VOG iodine sorbent systems should be conservatively modeled to accommodate a capacity of 40–50 mg I/g sorbent on AgZ to meet required decontamination factors.

## Data Availability

The raw data supporting the conclusion of this article will be made available by the authors, without undue reservation.
